# Editorial: Combinational therapy and nanotechnologies in combating pathogenic microbes and antibiotic resistance

**DOI:** 10.3389/fphar.2024.1406043

**Published:** 2024-05-07

**Authors:** Kwang-sun Kim

**Affiliations:** Department of Chemistry and Chemistry Institute for Functional Materials, Pusan National University, Busan, Republic of Korea

**Keywords:** multidrug-resistant pathogens, combinational therapy, nanocomposites, infectious diseases, tuberculosis

## Abstract

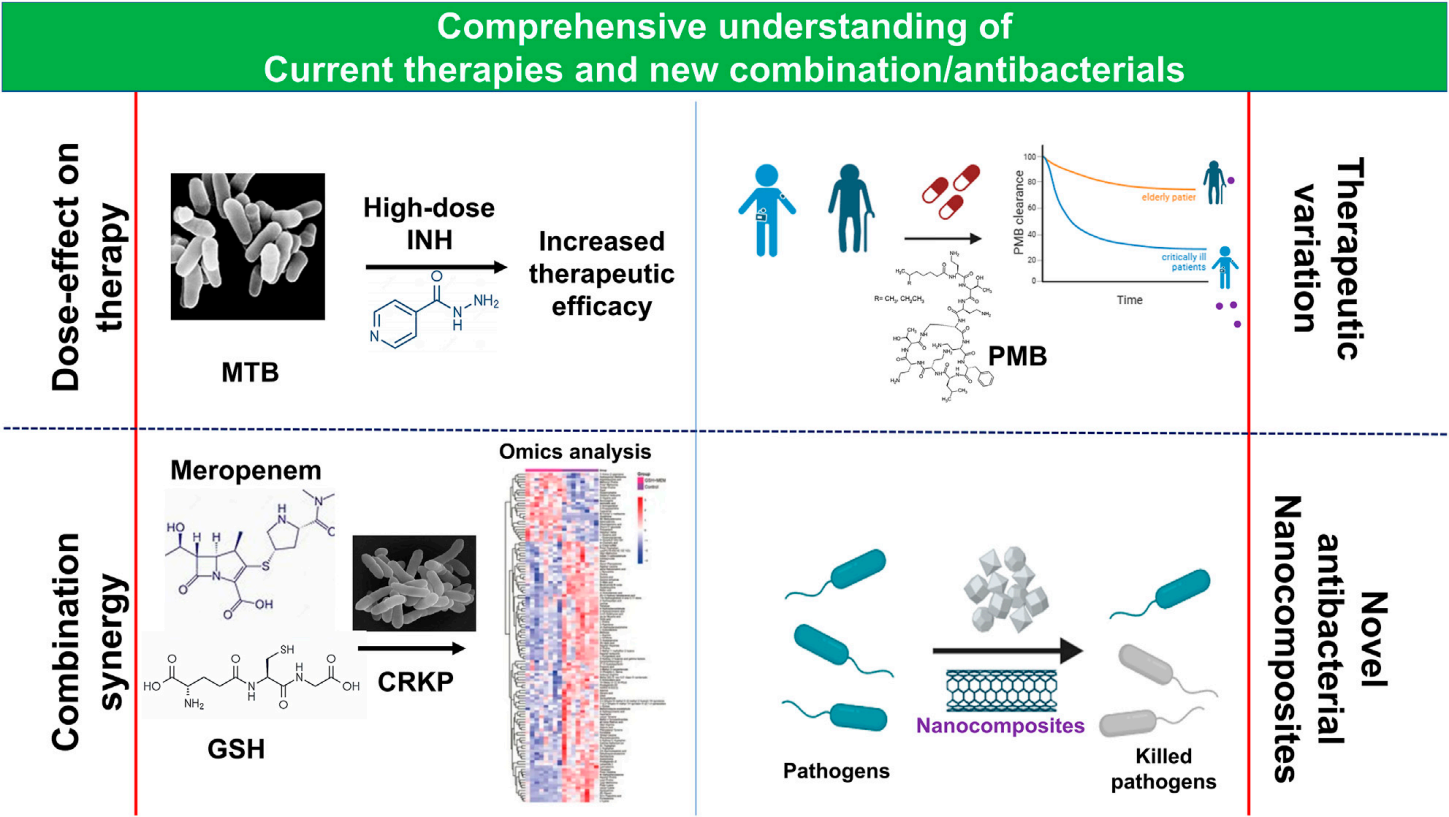

While antibiotics have significantly reduced bacterial infections and deaths, their indiscriminate use and environmental factors have resulted in multidrug resistance (MDR), which limits the effectiveness of current therapies. MDR is estimated to cause 700,000 deaths annually and could rise to 10 million by 2050 without immediate intervention (O’Neill, 2016). Developing new antibiotics is a potential solution, but the development pipeline is limited, and MDR evolves rapidly. To overcome these limitations, a comprehensive understanding of the limitations of current antibiotic therapy and new combinatorial strategies with multiple antibiotics and antimicrobials (Tyers and Wright, 2019) are needed.

The current topic explores the comparative pharmacokinetics (PK) of polymyxin B (PMB), a last-resort antibiotic for MDR Gram-negative bacterial infections, and a meta-analysis of high-dose isoniazid therapy for MDR or XDR *Mycobacterium tuberculosis* (MTB). Additionally, it introduces new combination therapies involving glutathione (GSH) and nanocomposites.

Carbapenem-resistant *Klebsiella pneumoniae* (CRKP) is a major contributor to nosocomial infections in humans, particularly those leading to hospital-acquired urinary, pneumonia, and bloodstream infections (Pendleton et al., 2013) and has been regarded as one of the critical-priority bacteria by the WHO (Tacconelli et al., 2018; Zhen et al., 2021). This drug-resistant bacterium is challenging to treat due to its resistance to multiple antibiotics like β-lactams, fluoroquinolones, and aminoglycosides. However, exogenous GSH, with its antibacterial properties and ability to clear biofilms, could be a potential solution (Das et al., 2019). GSH supports antibiotics like quinolones and aminoglycosides and enhances bacterial killing and impacts antibiotic effectiveness (Goswami et al., 2006; Goswami and Jawali, 2007). The study by Yi et al. (2023) demonstrated that GSH increases the potency of meropenem, a commonly used antibiotic for severe MDR Gram-negative pathogens, including CRKP (Truong et al., 2022). The authors determined the minimum inhibitory concentration (MIC) and minimum bactericidal concentration (MBC) of GSH against 30 CRKP isolates and found that 9 mg/mL of GSH effectively eradicated 99.9% of CRKP. Additionally, the synergy between GSH and meropenem was evaluated by determining the fractional inhibitory concentration index (FICI), and it was found that 86.7% of the isolates showed significant antagonism of bacterial growth after 24 h of exposure to the synergistic combination. The GSH-induced potency of meropenem was found to be concentration-dependent, and the underlying mechanisms were assessed by analyzing common antimicrobial mechanisms, including ROS generation and metabolite analysis. The study found that the increase in membrane permeability due to alterations in glycerophospholipids is the plausible mechanism of the synergy, which could potentially provide a new route for CRKP treatment.

Polymyxins are typically considered last-resort antibiotics against extensively drug-resistant (XDR) Gram-negative bacteria, with PMB being associated with a high rate of nephrotoxicity, believed to originate from its accumulation in the renal proximal tubule (Manchandani et al., 2015; Yun et al., 2015; Liu et al., 2023). While international guidelines recommend calculating the dose of PMB based on the patient’s weight, regardless of age (Tsuji et al., 2019), the relationship between age, illness status, and polymyxin-related nephrotoxicity remains controversial. To investigate PMB exposure in elderly and young critically ill patients and determine the covariates of PK for PMB in critically ill patients, Zeng et al. (2024) measured plasma PMB concentrations over a 24-hour period at steady state. Their results showed that total body weight, rather than age, was the primary factor affecting PMB clearance, consistent with prior studies. However, this study also revealed that elderly patients exhibited delayed PMB clearance and metabolism compared to young critically ill patients. This research is noteworthy as it is the first to compare PMB exposure and individual PK parameters in critically ill patients of different ages, given standard PMB dosing, and contributes to optimizing PMB use in clinical practice for critically ill patients. A limitation of this study, however, is its small sample size of critically ill patients with varying renal functions, ages, and body weights.

*Mycobacterium tuberculosis* continues to be a significant challenge as it is the leading cause of mortality globally (WHO, 2023). Despite its simplicity, the action mechanism of isoniazid (INH), the most efficient prophylactic drug against MTB infections since 1952 (Fernandes et al., 2017), is complicated (Unissa et al., 2016). Its continuous use can result in the emergence of MDR and XDR MTB due to acquired genetic mutations (Dominguez et al., 2023). Although the WHO previously recommended high-dose INH as an MDR and XDR MTB regimen (WHO, 2019), it was removed from standard treatment in recent WHO guidelines (WHO, 2020) due to insufficient efficacy data. However, high-dose INH is still indicated for children, patients without sufficient alternatives, and special mutants caused by low-level INH resistance (Dominguez et al., 2023). Some studies have reported the clinical efficacy of the regimen (Cambau et al., 2015; Lempens et al., 2018), but no recent systematic reviews and meta-analyses have been published on the clinical efficacy and safety outcomes of high-dose INH therapy. A study by Zhou et al. (2024) found that high-dose INH administration for MDR-MTB treatment is associated with excellent efficacy and a favorable outcome, with an acceptable adverse-event profile. However, more research is needed to investigate the impact of high-dose INH on long-term outcomes and its role in specific subpopulations.

Nanocomposites, made up of matrix materials and nanofillers, are a promising alternative to conventional antibiotics, as per a comprehensive review by Saravanan et al. (2023). These nanocomposites, including metal/metal oxides, chitosan-metals, titanium-based nanoparticles, graphene-based materials, and multi-walled carbon nanotubes with or without polymers, can serve as effective antibacterial agents when tailored to enhance treatment efficacy and reduce the risk of MDR. Furthermore, combining these nanocomposites with existing antibiotics can create novel antimicrobials that increase the efficacy of current antibiotics or enable the use of abandoned antibiotics due to resistance. However, challenges such as toxicity, safety, scalability, selectivity, and bioavailability need to be addressed before clinical application.

The studies discussed in this Research Topic will offer insights into current drug usage against MDR or XDR bacterial infections, materials for targeted bacterial killing, and the development of biocompatible and modality-specific antimicrobials.
